# A Cross-Cultural Exploratory Study of Health Behaviors and Wellbeing During COVID-19

**DOI:** 10.3389/fpsyg.2020.608216

**Published:** 2021-01-12

**Authors:** Montse C. Ruiz, Tracey J. Devonport, Chao-Hwa (Josephine) Chen-Wilson, Wendy Nicholls, Jonathan Y. Cagas, Javier Fernandez-Montalvo, Youngjun Choi, Claudio Robazza

**Affiliations:** ^1^Faculty of Sport and Health Sciences, University of Jyväskylä, Jyväskylä, Finland; ^2^Sport and Physical Activity Research Centre, University of Wolverhampton, Wolverhampton, United Kingdom; ^3^Faculty of Health, Education and Society, University of Northampton, Northampton, United Kingdom; ^4^Department of Sports Science, University of the Philippines Diliman, Diliman, Philippines; ^5^Centre for Health Research, University of Southern Queensland, Queensland, QLD, Australia; ^6^Faculty of Health Sciences, Universidad Pública de Navarra, Pamplona, Spain; ^7^Department of Physical Education, Jeju National University, Jeju, South Korea; ^8^BIND-Behavioral Imaging and Neural Dynamics Center, Department of Medicine and Aging Sciences, “G. d’Annunzio” University of Chieti-Pescara, Chieti, Italy

**Keywords:** coronavirus, inactivity, food consumption, behavior change, resources

## Abstract

This study explored the influence of the COVID-19 pandemic on perceived health behaviors; physical activity, sleep, and diet behaviors, alongside associations with wellbeing. Participants were 1,140 individuals residing in the United Kingdom (*n* = 230), South Korea (*n* = 204), Finland (*n* = 171), Philippines (*n* = 132), Latin America (*n* = 124), Spain (*n* = 112), North America (*n* = 87), and Italy (*n* = 80). They completed an online survey reporting possible changes in the targeted behaviors as well as perceived changes in their physical and mental health. Multivariate analyses of covariance (MANCOVA) on the final sample (*n* = 1,131) revealed significant mean differences regarding perceived physical and mental health “over the last week,” as well as changes in health behaviors during the pandemic by levels of physical activity and country of residence. Follow up analyses indicated that individuals with highest decrease in physical activity reported significantly lower physical and mental health, while those with highest increase in physical activity reported significantly higher increase in sleep and lower weight gain. United Kingdom participants reported lowest levels of physical health and highest increase in weight while Latin American participants reported being most affected by emotional problems. Finnish participants reported significantly higher ratings for physical health. The physical activity by country interaction was significant for wellbeing. MANCOVA also revealed significant differences across physical activity levels and four established age categories. Participants in the oldest category reported being significantly least affected by personal and emotional problems; youngest participants reported significantly more sleep. The age by physical activity interaction was significant for eating. Discussed in light of Hobfoll (1998) conservation of resources theory, findings endorse the policy of advocating physical activity as a means of generating and maintaining resources combative of stress and protective of health.

## Introduction

Pandemics are identified as large-scale outbreaks of infectious diseases. They significantly increase morbidity and mortality over an extensive geographic area and contribute to substantial economic, social, and political disruption (Madhav et al., 2017). The World Health Organization (World Health Organization, 2020) declared COVID-19 a global pandemic the likes of which have not been experienced in modern times (Kickbusch et al., 2020). In order to minimize the spread of the virus, many countries introduced confinement/lockdown measures including “stay at home” orders, closure of schools, businesses and places of congregation, and travel restrictions. During this time, people were allowed to leave their homes only to purchase essential items (e.g., food, medicines), seek essential treatment, go to work (only for jobs considered essential), or to assist and care for dependents. Given the severity of the pandemic and scale and duration of containment measures, there has been a call to examine potential changes in health behaviors during the COVID-19 pandemic along with mental health outcomes. It is of importance to examine both, as health behaviors are strongly associated with mental health (Mammen and Faulkner, 2013; Parletta et al., 2016).

This study examined sleep, eating, and physical activity outcomes during the COVID-19 pandemic along with physical and mental wellbeing. Previous literature has shown the importance of each health behavior and evidenced how they may be affected by pandemics. Associations with health and wellbeing for each health behavior will be examined followed by emerging literature specific to the context of the COVID-19 pandemic. Starting with sleep; sleep loss, alterations to sleep-wake timings, and/or increased night awakenings are known to compromise immune function (Besedovsky et al., 2012) as well as cause disruptions to a range of metabolic hormones that affect energy homeostasis (Spiegel et al., 2004, 2005; Capers et al., 2015). Increased susceptibility to the common cold has been evidenced following reduced sleep efficiency (Cohen et al., 2009) and an increased long-term risk of developing chronic diseases (von Ruesten et al., 2012). Inadequate sleep has also been shown to affect other health behaviors, for example, leading to poor dietary habits and reduced motivation to exercise (Spiegel et al., 2004, 2005; Kline, 2014; Capers et al., 2015). Sleep loss and disturbance may also exacerbate existing mental health problems via effects on affective functioning; which is inclusive of mood, emotion, and emotion regulation (Watling et al., 2017). The effects of sleep loss and disruption on mood may trigger and maintain a negative spiral leading to ongoing sleep problems and psychopathology (Pilcher and Huffcutt, 1996; Short and Louca, 2015).

The containment measures of COVID-19 have the potential to influence sleep behaviors. There is the possibility of sleep disruptions due to emotional responses to the pandemic (e.g., stress, anxiety, and depression) while, on the other hand, the ‘stay at home’ mandate may provide increased flexibility in sleep-wake timings and sleep duration. Early findings regarding sleep during COVID-19 evidence both detriments and gains to sleep quantity and quality. Fitbit (2020) revealed that people globally appear to be going to bed later and achieving more sleep than usual since the COVID-19 outbreak. However, a survey completed by 2,254 United Kingdom respondents revealed that 38% reported sleeping less or less well than prior to the country being placed on lockdown (Kings College London (KCL), 2020). Within the same survey, 49% of respondents reported feeling more anxious and depressed as a direct result of COVID-19. Gupta et al. (2020) examined changes in sleep pattern and sleep quality during COVID-19 using a questionnaire distributed via social media platforms. Compared to the pre-lockdown period, all occupational groups (except health professionals) evidenced a shift to a later bedtime, a reduction in night-time sleep, an increase in day-time napping, and a deterioration in sleep quality. Reductions in sleep duration were associated with depressive symptoms. Collectively, these findings offer support for the contention that sleep disruption may be associated with emotional responses to the pandemic.

With regards to eating, research has identified several reasons for modified eating behaviors including opportunity induced eating and coping with unpleasant emotions (Verhoeven et al., 2015). Emotional eating is defined as (over) eating in response to emotions including depression, irritability, stress, or anxiety (van Strien et al., 2013; Frayn et al., 2018; van Strien, 2018), with food consumption used to regulate emotions (Nicholls et al., 2015; Devonport et al., 2017). For example, snacking behavior may be elicited by emotions such as boredom or stress with snacking providing a distraction from anxiety or relief from boredom. Food may also be used to connect with others, form part of celebrations or be used as a reward or a treat (Locher et al., 2005). Emotional eating that leads to increased calorie consumption over time can lead to obesity and associated health concerns (Micanti et al., 2017; van Strien, 2018).

Since the outbreak of COVID-19, panic buying and stockpiling of household items in response to “stay at home” mandates have been well reported. On the one hand, this may support the preparation of more home cooked meals and consumption of fewer preservatives/additives. On the other hand, this may increase snacking behaviors driven by opportunity induced eating, increased screen/sedentary time and an increase in unpleasant emotions such as stress, anxiety, and boredom. Di Renzo et al. (2020) undertook an online survey between 24 April and 18 May, 2020 among Italian participants (when confinement measures were in place). They found that a high percentage of respondents experienced a depressed mood, anxious feelings, hypochondria and insomnia (61.3, 70.4, 46.2, and 52.2%, respectively). Almost half felt anxious due to their eating habits, consumed comfort food and were inclined to increase food intake to feel better. A systematic review and meta-analysis of literature examining the prevalence of stress, anxiety and depression among the general population during COVID-19 (Salari et al., 2020) found evidence of an increase in each unpleasant emotion during the pandemic. There has been a call for further investigation of eating behaviors during COVID-19 with larger population studies (Di Renzo et al., 2020). Following the notion that unpleasant emotion may result in eating behaviors, there is reason to contend that people may consume more food during the pandemic. Finally, the benefits of physical activity for physical and emotional health are well established (Mammen and Faulkner, 2013; Pedersen and Saltin, 2015). For example, Mammen and Faulkner (2013) identified physical activity as an important protective factor in reducing the risk of developing depression. Physical activity strengthens the immune system (Hojman, 2017) and is effective in preventing and treating non-communicable conditions, such as heart disease and diabetes (Powell et al., 2018). Such conditions lead to poorer outcomes among those contracting COVID-19 (Chen et al., 2020; Ma and Holt, 2020). In addition to direct health benefits, physical activity is known to affect other key health behaviors. For example, a systematic review highlights the positive role of both acute and regular exercise on sleep quality (Kredlow et al., 2015). Sallis and Pratt (2020, p. 2) advocate that “due to its multiple benefits, physical activity should not be an afterthought during this pandemic. Being active should be a key recommendation.” Indeed, when first introducing containment measures, many governments, encouraged individuals to exercise with mandates around where and how often. Pertinent to the present study, this included Finland, Latin America, North America, and the United Kingdom. This promotion of exercise while concurrently emphasizing social distancing measures indicates that exercise was recognized and advocated as a means of maintaining physical and mental wellbeing during the pandemic (Matias et al., 2020).

Despite physical activity being encouraged in many countries, for many, social distancing measures, increased screen time, lost incidental physical activity (such as walking to and for work), and the closure of facilities including gyms, parks, and sports facilities may have led to reduced levels of physical activity (Chen et al., 2020). Particularly when combined with the increased possibility of sedentary behaviors (sitting, reclining, TV viewing, using mobile devices, or playing video games) resulting from prolonged homestay. For example, Di Sebastiano et al. (2020) used a national physical activity tracking app to determine device-measured physical activity levels among 2,338 Canadians to determine changes in physical activity four weeks pre-pandemic and six weeks post-pandemic declaration. Although moderate-to-vigorous physical activity returned to pre-pandemic levels, significant and sustained declines in incidental light physical activity and steps were observed indicating a sustained loss of incidental physical activity.

Wearable activity trackers provide a useful measure of population physical activity and correlate highly with actual steps in experimental conditions (Evenson et al., 2015), so these data could be used to provide an indicator of activity during the pandemic. Fitbit (2020) reported a decline in step count across every country examined during the week of 22 March, 2020 compared to the same week in 2019, with European countries showing the most dramatic decline ranging from 7% (Germany) to 38% (Spain). Garmin (2020b) documented a 12% decline in total worldwide steps during April 2020, with worldwide steps from workout activities increasing by 24%. Garmin (2020a) data also evidenced a transition to indoor activities such as indoor cycling and running when comparing the five weeks prior to and after March 9th (where national lockdowns started to take effect in Italy). In Spain and Italy, where countrywide lockdowns were most extreme during this period, outdoor running dropped significantly with respective decreases of 68 and 42%. A switch to indoor running was evident, with country increases of 130, 84, and 18% for Italy, Spain, and France, respectively. Indoor cycling showed an increase of 157, 273, and 309% in France, Spain, and Italy respectively; steep increases in an activity that typically decreases during this time. When undertaking indoor cycling, many used platforms such as Zwift allowing them to connect with others worldwide and ride together. It must be borne in mind that those sufficiently motivated to invest in tracking equipment such as Fitbit and Garmin may form part of a habitually active community, and so physical activity data from a representative sample is required. When examining emerging effects of COVID-19 on mental wellbeing, stressors resulting from the pandemic include infection fears posed by COVID-19, social distancing requirements, boredom, frustration, inadequate supplies, inadequate information, media reporting negatively skewed number of people infected and resultant deaths, financial loss, job loss, and stigma (Brooks et al., 2020; Garfin et al., 2020; Hamel et al., 2020; Nelson et al., 2020; Van Lancker and Parolin, 2020). Wang et al. (2020) conducted a cross-sectional study with more than 1,000 Chinese adults, and found that emotional disturbance was evident during the pandemic. This included experiencing depression (13.8% mild depression symptoms, 12.2% moderate depression, and 4.3% severe depression), stress (24.1% reported suffering from mild stress while 8.1% reported moderate or severe stress levels) and anxiety (7.5% mild, 20.4% moderate, and 8.4% severe). Furthermore, requirements for social distancing have hindered access to a commonly used and effective coping strategy; social support. Support provided from others can help individuals cope with stress, regulate emotions, attain informational and practical assistance, and remain resilient during difficult times (Greenglass, 1993; Jetten et al., 2017; Williams et al., 2018). As such, it is expected that citizens from COVID-19-afflicted countries would experience similar deleterious effects on mental wellbeing (Wang et al., 2020).

Hobfoll’s conservation of resources (COR; Hobfoll, 2011) theory posits that individual resources connect with quality of life and wellbeing. Resources and their growth associate with a higher quality of life, while their loss is associated with lower quality of life. However, “resource loss is disproportionately more salient than is resource gain” (Hobfoll, 1998, p. 62). Stress occurs in situations that exceed the capabilities of resources, threaten them, lead to their exhaustion, or when resources do not generate the intended effects (Hobfoll et al., 2018). In fact, research has shown that resource loss is associated with deleterious outcomes above and beyond the effects of the trauma itself (Hobfoll, 2011). Investing in resources to protect against resource loss, recover from losses, and gain resources is of importance (Hobfoll, 1998). Individuals who are able to generate, accumulate, and maintain resources are less likely to experience resource loss, and as such are more likely to experience a sense of success and gain, leading to life satisfaction (Zhou and Lin, 2016). Those with more resources are less vulnerable to reduced mental wellbeing, while those who lack resources are more likely to experience a loss spiral whereby a continuous loss cycle is entered (Hobfoll, 1998; Hobfoll and Shirom, 2001).

There is an increased risk of loss spirals in situations of chronic stress, such as that presented by COVID-19. Resources may include external resources such as social support, mobility, co-parenting, finance, as well as internal resources such as physical health, self-efficacy, emotional intelligence, or knowledge. In the context of the present study, the health behaviors of sleep, eating, and physical activity present an investment in resources supportive of physical and mental wellbeing. Studies released to date indicate that for many, the pandemic and associated containment measures have required radical lifestyle changes that disrupted usual daily activities (Jiménez-Pavón et al., 2020). We contend that for some, this has resulted in maladaptive changes in health behaviors including reduced or altered physical activity behaviors, increased food consumption, and disturbed sleep (Di Renzo et al., 2020; Di Sebastiano et al., 2020; Kings College London (KCL), 2020), and presented consequences for resource accumulation and maintenance, and thus mental health (Liu et al., 2020; Salari et al., 2020; Sun et al., 2020; Wang et al., 2020). Examining the health behaviors and wellbeing outcomes of individuals during the COVID-19 pandemic could help identify groups most vulnerable to loss spirals, and reduced health and wellbeing during similar eventualities. In particular, understanding short and long-term effects, along with the interactions of health behaviors with mental health could inform the deployment of resources in future eventualities with shared characteristics.

The social context has an important effect on pandemic response (Van Bavel et al., 2020), so variation in response between countries is of interest, and exploring this can inform future response planning and target interventions appropriately. With reference to the COR theory (Hobfoll, 2011), there is good justification to expect variation between countries, as those with prior experience of lockdown, through epidemics such as SARS, may have been able to call upon previously developed resources to engage effective and practiced coping strategies, and so experience better outcomes in terms of their wellbeing. Brooks et al. (2020) called for a need to better understand cultural differences in the psychological impact of quarantine. However, emerging research on response to the COVID-19 pandemic is tending toward single country samples, for example, Italy (Landi et al., 2020), China (Liu et al., 2020), Spain (Rodríguez-Rey et al., 2020b), and Netherlands (van Tilburg et al., 2020), which offers limited opportunity to explore the possible influence of cross-cultural variation. Thus, generalization and application of research findings internationally can only be done with caution.

The present study aimed to examine health behaviors and wellbeing nationally, with a view to exploring the emerging trends between a selection of countries with different confinement measures. Based on the literature reviewed, we focused on examining potential changes in physical activity as well as eating, sleep, health, and wellbeing. Drawing on Hobfoll (2011) conservation of resources theory, we expect that participants experiencing strict containment measures during the COVID-19 pandemic will experience a loss of resources, which will be associated with reduced physical and emotional health. Our first aim was to examine potential differences in individuals’ perceived health behaviors during the COVID-19 pandemic (i.e., eating and sleep) and wellbeing across countries and changes in physical activity. In line with the extensive body of work on the benefits of physical activity (Mammen and Faulkner, 2013; Pedersen and Saltin, 2015; Hojman, 2017; Powell et al., 2018), we expected exercise to be associated with less detrimental consequences of the pandemic on health behaviors. Due to the variability in the containment measures put into place to slow the spread of the virus in different countries or regions, significant differences in the study variables were expected, with individuals experiencing most strict restrictions perceiving most detrimental changes in health behaviors. Because COVID-19 is considered most deadly among older people (United Nations, 2020), our second aim was to examine differences in the targeted health behaviors by age groups. Due to the novelty of the virus, no specific hypotheses were drawn regarding age comparisons.

## Materials and Methods

A web-based survey was prepared using the Qualtrics platform, which allowed gathering information from an international sample of individuals during the COVID-19 pandemic period.

### Participants

An initial sample of 1,458 participants took part in the study, from which 1,200 were valid completed surveys. Participants resided in United Kingdom and Ireland (*n* = 230), South Korea (*n* = 204), Finland (*n* = 171), Philippines (*n* = 132), Latin America (*n* = 124), Spain (*n* = 112), North America (*n* = 87), and Italy (*n* = 80). Selection of countries^1^ targeted cultural diversity and their status in terms of response to the pandemic. Sixty participants (4.8%) indicated living in a different country at the time of the study. Due to different restrictions put into place in different countries, data from participants living in other countries than the ones targeted were excluded. Participants’ age ranged from 18 to 76 years (*M* = 33.91, *SD* = 12.88). More than half of the participants^2^ were female (*n* = 745; 65%), single (*n* = 598; 53%), and living with two or more household members (*n* = 671; 59%). Most participants reported living in a detached house or family house with a garden or yard space (*n* = 425; 37%), while 18% of participants reported living in apartments without a balcony, terrace or private garden.

### Measures

The survey assessed participants’ physical and mental health, as well as changes in sleep, eating, exercising, and wellbeing during the COVID-19 pandemic.

#### Demographic Information and Current Living Situation

Participants were asked to provide demographic information including their age, gender, country of residence, and relationship status. Their current living situation was assessed with questions relating to their perception of being in lockdown or not and living arrangements (i.e., type of property and household members they live with). Varied responses to the pandemic across and within countries, and the differential implications determined by factors such as occupation (keyworker status), age and region, presented a rationale for using an individual’s *perception* of whether or not they were in lockdown at the point of completing the survey. This personal perception is important, as individuals with job roles deemed essential in running health and community services (e.g., front line health workers, postal services, refuse collection, and food outlets) continued working as normal, and so may have not self-defined as being in lockdown. Conversely, while Finland had no mandated lockdown, individuals may have chosen to self-isolate and so perceived themselves to be so. This personal perception, therefore, provided more precise information on individual experience than applying the countrywide status to that individual.

#### Physical and Mental Health

Two items from the SF-8 Health Survey (Ware et al., 2001) were used as indicators of physical and mental health. The items typically ask participants to rate their health in considering how they have felt during the previous month. Due to the rapidly changing conditions caused by the pandemic, the time frame was delimited to perceptions during the previous week. To measure participants’ physical health, the following item “Overall, how would you rate your health during the past week?” was rated on a six-point scale (1 = *very poor*, 6 = *excellent*). As indicator of mental health, using the same timeframe of “during the past week,” a second item asked participants to assess “How much did personal or emotional problems keep you from doing your usual work, school or other daily activities?”, which used the following anchors: 1 (*not at all*), 2 (*very little*), 3 (*somewhat*), 4 (*quite a lot*), and 5 (*could not do daily activities*). Previous research has demonstrated that the SF-8 Health Survey is a feasible, reliable, and valid instrument, which is widely used with translations to over 30 languages (Yiengprugsawan et al., 2014).

#### Perceived Changes in Working Situation, Health Routines, and Wellbeing

Participants were asked to report changes in their working situation indicating whether their workload had increased, decreased, or remained the same, if they worked from home, were laid-off, or unemployed as a consequence of the COVID-19 pandemic. They also reported possible changes in their basic health routines (i.e., eating, sleeping, and exercising), weight, and wellbeing. These changes were assessed on an 11-point scale (−5 = *significant decrease*, 0 = *no change*, +5 = *significant increase*). Single item measures with high face validity, as in this case, have received strong support (Bowling, 2005; Tenenbaum et al., 2007) and are deemed appropriate in situations where reducing respondent burden and administration simplification is required. Previous research has demonstrated the appropriateness of single item measures in the assessment of changes in physical activity (Portegijs et al., 2017; O’Halloran et al., 2020), restrictive eating (Haynos and Fruzzetti, 2015), and mental health (Ahmad et al., 2014).

### Procedure

Separate web-based surveys were constructed using the Qualtrics platform, including all information and questions in the native languages of the targeted countries. On the first page of the survey, individuals were informed of the nature and purpose of the study and what their participation would entail. They were also informed of their right to withdraw from the study. Participants were asked for their informed consent to continue, which was granted electronically. This study received ethical approval from the University of Wolverhampton ethics committee (Unique code: 01/20/AF1/UOW). The research was launched and distributed using social media platforms, with colleagues located in targeted countries asked to disseminate the project. The survey links were distributed online via social networking sites such as Facebook, Twitter, and LinkedIn. As an example of reach, there were 27,532 twitter impressions for announcements of the United Kingdom survey from one co-author alone. One inclusion criterion applied; that the participant was required to be over 18 years of age. Completion of the web-based survey took approximately 10 min.

### Data Analysis

Prior to the main analysis, data were screened for missing values, potential outliers, and violations of assumptions of normality, linearity, multicollinearity, and homoscedasticity (Tabachnick and Fidell, 2019). Descriptive statistics and Pearson product-moment correlation coefficients were computed for all studied variables (see Supplementary Tables S2, S3). Four groups were created based on quartile splits for different levels of physical activity during the COVID-19 pandemic and age groups. Two multivariate analyses of covariance (MANCOVAs) were conducted. The first one examined group differences considering change in Physical Activity by Country and the second considering Physical Activity by Age. Perceived physical and mental health, change in health routines (i.e., sleep and eating), weight, and wellbeing during the COVID-19 pandemic were the outcome variables. Perception of lockdown (yes vs no), working situation (employed vs unemployed or laid off), and household number (one/two/more than three) were entered as covariates. Bonferroni adjustment for multiple comparisons was used.

## Results

### Descriptive Statistics

**FIGURE 1:**
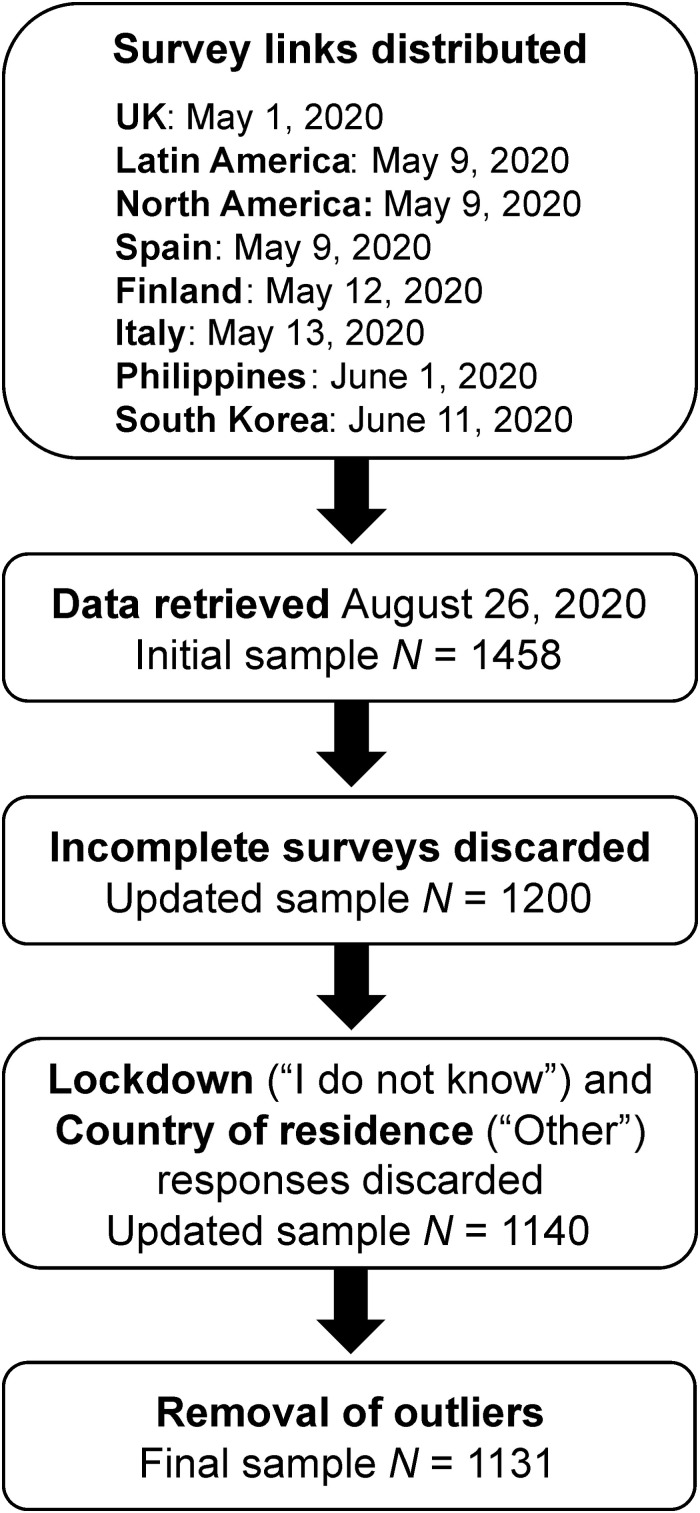
Flow diagram illustrating the data collection process and data used in the analyses.

Data screening suggested the removal of nine cases identified as outliers (Mahalanobis’ distance, *p* < 0.001). The final sample consisted of 1,131 participants (see Figure 1). Group comparisons across Physical Activity levels and Country of residence will be presented first, followed by Physical Activity by Age comparisons. Four groups of participants were identified based on reported physical activity as follows: very inactive (*n* = 359, −3.24 ± 1.14), inactive (*n* = 294, −0.32 ± 0.47), active (*n* = 328, 2.17 ± 0.79), and very active (*n* = 150, 4.57 ± 0.50). The number of participants by country of residence is reported in the Participants section. The following four age groups were identified: 18–23 years (*n* = 326), 24–30 years (*n* = 302), 31–43 years (*n* = 323), and older than 43 years (*n* = 298).

### Differences Across Reported Physical Activity and Country

Differences in participants’ health behaviors and wellbeing were first examined across participants’ reported changes in physical activity and country of residence. The results of the MANCOVA indicated significant main effects of Physical Activity, Wilk’s λ = 0.768, *F*(18, 3,094) = 16.544, *p* < 0.001, η_*p*_^2^ = 0.084, and Country, Wilk’s λ = 0.820, *F*(42, 5,040) = 5.197, *p* < 0.001, η_*p*_^2^ = 0.033. The Physical Activity by Country interaction was also significant, Wilk’s λ = 0.858, *F*(126, 6,594) = 1.325, *p* = 0.009, partial η_*p*_^2^ = 0.025. Two of the covariates were significant, perception of being or not in lockdown, Wilk’s λ = 0.979, *F*(6, 1,074) = 3.785, *p* < 0.001, η_*p*_^2^ = 0.021, and being or not employed, Wilk’s λ = 0.975, *F*(6, 1,074) = 4.634, *p* < 0.001, η_*p*_^2^ = 0.025. Same significant results were obtained after excluding the covariates.

*Post hoc* analysis on the main effects of Physical Activity indicated that individuals reporting highest increases in physical activity rated significantly higher mean scores for physical health perceived “during the last week” and wellbeing compared to individuals reporting higher decrease in physical activity (see Table 1 for pairwise comparisons). Individuals who reported highest decrease in physical activity rated significantly higher scores assessing emotional impact “during the last week,” reflecting lowest mental health compared to the rest of participants. Individuals reporting highest decrease in physical activity also reported significantly higher scores for eating compared to individuals with a moderate decrease in physical activity. Significant differences were also found in regard to sleep, with individuals reporting highest increase in physical activity indicating more sleep than those with highest decrease in physical activity. Individuals with highest decrease in physical activity reported significantly higher weight gains compared to the rest of participants.

**TABLE 1:** Descriptive statistics and pairwise comparisons considering reported changes in participants’ physical activity.

		Descriptive statistics			Pairwise comparisons			
Variables	Physical activity	M	SD	N	Very inactive	Inactive	Active	Very active
Physical health during last week	Very inactive	3.42	1.05	359	–			
	Inactive	3.91	1.00	294	**	–		
	active	3.94	1.03	328	**	n.s.	–	
	Very active	4.42	1.04	150	**	**	**	–
Emotional impact during last week	Very inactive	2.97	1.10	359	–			
	Inactive	2.28	1.11	294	**	–		
	Active	2.47	1.09	328	**	n.s.	–	
	Very active	2.23	1.05	150	**	n.s.	n.s.	–
Eating	Very inactive	1.34	2.24	359	–			
	Inactive	0.70	1.58	294	**	–		
	Active	1.17	1.94	328	n.s.	*	–	
	Very active	1.07	2.21	150	n.s.	n.s.	n.s.	–
Sleep	Very inactive	0.27	2.86	359	–			
	Inactive	0.19	1.98	294	n.s.	–		
	Active	0.88	2.33	328	*	*	–	
	Very active	1.05	2.65	150	*	*	n.s.	–
Weight	Very inactive	1.63	2.31	359	–			
	Inactive	0.89	1.62	294	**	–		
	Active	0.55	2.02	328	**	n.s.	–	
	Very active	0.09	2.32	150	**	*	n.s.	–
Wellbeing	Very inactive	−1.46	1.99	359	–			
	Inactive	−0.26	1.70	294	**	–		
	Active	−0.02	1.97	328	**	n.s.	–	
	Very active	1.44	2.70	150	**	**	**	–

**p < 0.001, *p < 0.05, n.s., non-significant.

**FIGURE 2:**
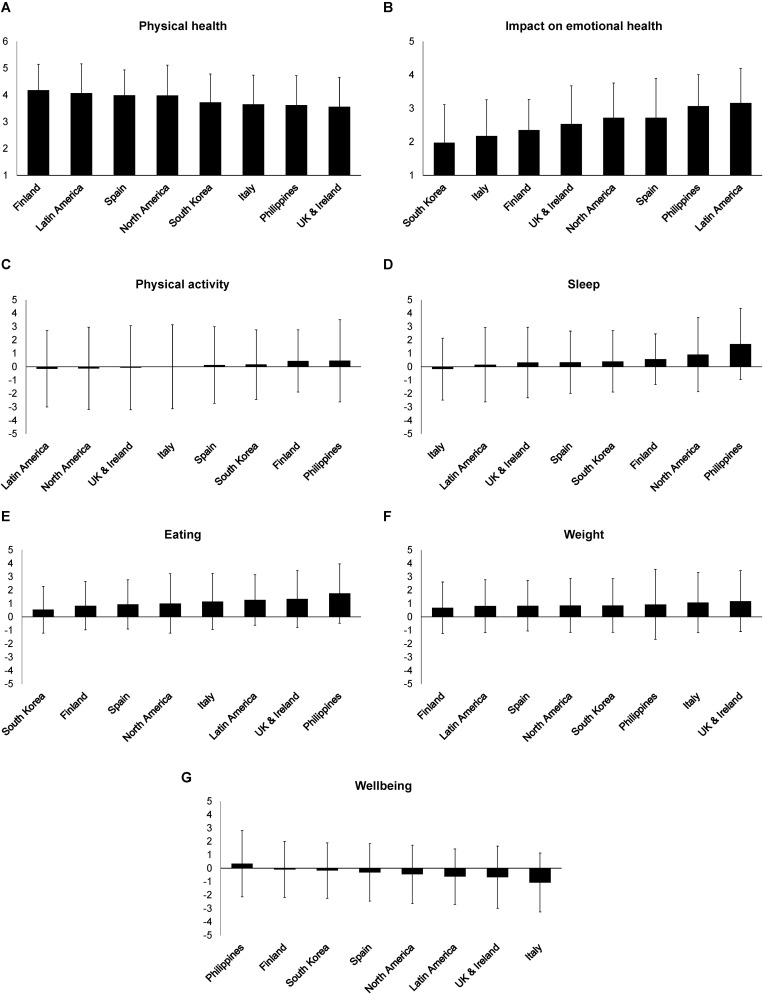
Means and standard deviations of the reported physical **(A)** and emotional **(B)** health perceived over the last week, reported changes in physical activity **(C)**, sleep **(D)**, eating behaviors **(E)**, weight **(F)**, and wellbeing **(G)** during the COVID-19 pandemic across countries (*N* = 1,131).

*Post hoc* analysis on the main effects of Country revealed significant mean differences in ratings of physical health perceived “during the last week” (see Table 2 for pairwise comparisons). Mean scores for United Kingdom participants were the lowest and significantly different from participants from Finland, who reported highest mean scores (see Figure 2). Regarding mental health perceived “during the last week,” significant mean score differences were found in ratings of Latin American participants who indicated being most affected by personal or emotional problems, while South Koreans reported being significantly least affected compared to most countries. Participants from the Philippines reported significantly higher mean scores for both sleep and eating behaviors compared to most countries. There were no significant differences in mean scores of changes in weight across the countries, with large variability ranging from ±1.90 (Spain) to ±2.61 (Philippines). In regard to perceived changes in wellbeing, Italian participants reported significantly lower mean scores compared to participants from Philippines and Finland, who reported highest scores.

**TABLE 2:** Descriptive statistics and pairwise comparisons across country.

		Descriptive statistics			Pairwise comparisons							
Variables	Region	M	SD	N	United Kingdom	South Korea	Finland	Philippines	Latin America	Spain	North America	Italy
Physical health during last week	United Kingdom	3.57	1.10	230	–							
	South Korea	3.73	1.06	201	n.s.	–						
	Finland	4.18	0.97	171	**	*	–					
	Philippines	3.63	1.10	129	n.s.	n.s.	**	–				
	Latin America	4.07	1.09	123	*	n.s.	n.s.	*	–			
	Spain	3.99	0.94	112	*	n.s.	n.s.	n.s.	n.s.	–		
	North America	3.99	1.13	86	*	n.s.	n.s.	n.s.	n.s.	n.s.	–	
	Italy	3.66	1.09	79	n.s.	n.s.	*	n.s.	n.s.	n.s.	n.s.	–
Emotional impact during last week	United Kingdom	2.53	1.14	230	–							
	South Korea	1.98	1.14	201	**	–						
	Finland	2.35	0.92	171	n.s.	*	–					
	Philippines	3.07	0.94	129	**	**	**	–				
	Latin America	3.16	1.04	123	**	**	**	n.s.	–			
	Spain	2.72	1.17	112	n.s.	**	n.s.	n.s.	*	–		
	North America	2.72	1.04	86	n.s.	**	n.s.	n.s.	n.s.	n.s.	–	
	Italy	2.18	1.08	79	n.s.	n.s.	n.s.	**	**	*	*	–
Eating	United Kingdom	1.35	2.13	230	–							
	South Korea	0.55	1.75	201	*	–						
	Finland	0.85	1.81	171	n.s.	n.s.	–					
	Philippines	1.76	2.22	129	n.s.	**	*	–				
	Latin America	1.28	1.89	123	n.s.	*	n.s.	n.s.	–			
	Spain	0.95	1.84	112	n.s.	n.s.	n.s.	*	n.s.	–		
	North America	1.01	2.22	86	n.s.	n.s.	n.s.	n.s.	n.s.	n.s.	–	
	Italy	1.15	2.09	79	n.s.	n.s.	n.s.	n.s.	n.s.	n.s.	n.s.	–
Sleep	United Kingdom	0.33	2.63	230	–							
	South Korea	0.41	2.30	201	n.s.	–						
	Finland	0.58	1.89	171	n.s.	n.s.	–					
	Philippines	1.71	2.66	129	**	**	*	–				
	Latin America	0.15	2.78	123	n.s.	n.s.	n.s.	**	–			
	Spain	0.35	2.32	112	n.s.	n.s.	n.s.	*	n.s.	–		
	North America	0.92	2.78	86	n.s.	n.s.	n.s.	n.s.	n.s.	n.s.	–	
	Italy	−0.16	2.31	79	n.s.	n.s.	n.s.	**	n.s.	n.s.	n.s.	–
Weight	United Kingdom	1.18	2.28	230	–							
	South Korea	0.86	2.00	201	n.s.	–						
	Finland	0.68	1.92	171	n.s.	n.s.	–					
	Philippines	0.94	2.61	129	n.s.	n.s.	n.s.	–				
	Latin America	0.81	1.98	123	n.s.	n.s.	n.s.	n.s.	–			
	Spain	0.85	1.90	112	n.s.	n.s.	n.s.	n.s.	n.s.	–		
	North America	0.86	2.01	86	n.s.	n.s.	n.s.	n.s.	n.s.	n.s.	–	
	Italy	1.09	2.25	79	n.s.	n.s.	n.s.	n.s.	n.s.	n.s.	n.s.	–
Wellbeing	United Kingdom	−0.68	2.32	230	–							
	South Korea	−0.17	2.07	201	n.s.	–						
	Finland	−0.08	2.09	171	n.s.	n.s.	–					
	Philippines	0.35	2.48	129	*	n.s.	n.s.	–				
	Latin America	−0.62	2.07	123	n.s.	n.s.	n.s.	*	–			
	Spain	−0.30	2.14	112	n.s.	n.s.	n.s.	n.s.	n.s.	–		
	North America	−0.45	2.18	86	n.s.	n.s.	n.s.	n.s.	n.s.	n.s.	–	
	Italy	−1.06	2.20	79	n.s.	n.s.	*	**	n.s.	n.s.	n.s.	–

**p < 0.001, *p < 0.05, n.s., non-significant.

Follow-up analysis revealed significant Physical activity by Country interaction for the participants’ perceived changes in their wellbeing (*p* = 0.049, η_*p*_^2^ = 0.030). Significant differences were found in wellbeing scores for Filipino participants who reported highest decrease in physical activity compared to the rest of countries studied. Filipinos also showed lowest decrement in wellbeing during the pandemic. In addition, wellbeing scores for Italian participants reporting a moderate increase in physical activity were significantly lower compared to Finnish (*p* = 0.003), Koreans (*p* = 0.003), North Americans (*p* = 0.048) and Filipinos (*p* = 0.029).

### Differences Across Reported Physical Activity and Age

MANCOVA yielded significant differences by Age, Wilk’s λ = 0.930, *F*(18, 3,083) = 4.437, *p* < 0.001, η_*p*_^2^ = 0.024, and Physical activity by age interaction, Wilk’s λ = 0.928, *F*(54, 5,562) = 1.513, *p* = 0.009, η_*p*_^2^ = 0.012. Two of the covariates were significant, Perception of lockdown (i.e., yes vs no), Wilk’s λ = 0.913, *F*(6, 1,090) = 17.384, *p* < 0.001, η_*p*_^2^ = 0.087, and Working situation (i.e., being employed vs not being employed), Wilk’s λ = 0.981, *F*(6, 1,090) = 3.523, *p* = 0.002, η_*p*_^2^ = 0.019, while the effect of Number of household members was not significant. Same significant results were obtained after excluding the covariates.

*Post hoc* analyses on the main effects of Age revealed significant differences in mental health with participants older than 31 years (i.e., 31–43 and >43) reporting being least affected by personal or emotional problems compared to their youngest counterparts (see Table 3). Participants in the youngest age category (i.e., 18–23) reported a significantly higher increase in sleep compared to older than 31 years of age participants. There were no significant differences for comparisons across age groups for physical health perceived “during the last week,” changes in weight, or wellbeing.

**TABLE 3:** Descriptive statistics and pairwise comparisons across age groups.

		Descriptive statistics			Pairwise comparisons			
Variables	Age groups	M	SD	N	18–23	24–30	31–43	>43
Physical health during last week	18–23	3.78	1.15	310	–			
	24–30	3.82	1.14	270	n.s.	–		
	31–43	3.78	1.02	271	n.s.	n.s.	–	
	>43	3.95	0.99	280	n.s.	n.s.	n.s.	–
Emotional impact during last week	18–23	2.79	1.18	310	–			
	24–30	2.67	1.14	270	n.s.	–		
	31–43	2.44	1.06	271	*	n.s.	–	
	>43	2.26	1.06	280	**	**	n.s.	–
Eating	18–23	1.06	2.25	310	–			
	24–30	1.22	2.05	270	n.s.	–		
	31–43	1.05	1.99	271	n.s.	n.s.	–	
	>43	1.03	1.69	280	n.s.	n.s.	n.s.	–
Sleep	18–23	1.10	2.78	310	–			
	24–30	0.60	2.53	270	n.s.	–		
	31–43	0.25	2.31	271	**	n.s.	–	
	>43	0.10	2.16	280	**	n.s.	n.s.	–
Weight	18–23	0.77	2.11	310	–			
	24–30	0.90	2.25	270	n.s.	–		
	31–43	1.08	2.29	271	n.s.	n.s.	–	
	>43	0.95	1.85	280	n.s.	n.s.	n.s.	–
Wellbeing	18–23	–0.36	2.36	310	–			
	24–30	–0.49	2.33	270	n.s.	–		
	31–43	–0.39	2.25	271	n.s.	n.s.	–	
	>43	–0.15	1.91	280	n.s.	n.s.	n.s.	–

**p < 0.001, *p < 0.05, n.s., non-significant.

Follow-up analysis revealed significant Physical activity by Age group interaction for eating (*p* = 0.003, η_*p*_^2^ = 0.022). Significantly higher eating scores (*p* < 0.001) were observed for the youngest participants (i.e., 18–23) with high increases in physical activity compared to participants in older age categories (i.e., 31–43 and >43, *p* = 0.001, *p* < 0.001, respectively).

Comparisons in the outcome variables for age groups and country could not be calculated because of the imbalanced number of participants in each group (e.g., 88% of Spanish participants were older than 30, while 77% of South Koreans were younger than 31). Supplementary Figure S1 presents means and standard deviations for the outcome variables for participants across countries and age groupings. Differences across gender were not calculated for the same reason (i.e., 32% of the total of participants were male).

## Discussion

The current study examined the influence of the COVID-19 pandemic on physical and mental health perceived “over the last week,” along with changes in physical activity, sleep, eating, body weight, and wellbeing. This was based on self-reported data from individuals residing in seven countries and Latin America. Overall, changes in physical activity during the pandemic were significant for participants’ health (physical and mental) and wellbeing. There was great variability in participants’ physical activity, with some individuals experiencing a high increase while others reporting a great decrease (Figure 2).

Comparisons between physical activity and country of residence, and between physical activity and age suggested that increased physical activity was related to less detrimental consequences of the pandemic on perceived physical and mental health. Perceptions of increased physical activity associated with better physical health as rated “over the past week,” along with increased wellbeing and sleep as compared to those perceiving a reduction in physical activity (Table 1). In contrast, decreased physical activity was related to worse mental health (represented by more personal and emotional problems), and a significant increase in eating and weight. Differences between increased exercise and perceived changes in eating were highly variable dependent on age and country of residence.

While the benefits of engaging in physical activity are widely accepted, it may hold increased value during scenarios such as pandemics where, as evident in the present study, the possibility of detriment to physical and mental health are increased. As findings indicate, physical activity seemed to buffer the deleterious effects of stressors. The mechanisms for this are subject to ongoing investigation, with contemporary research suggesting that improved mood post exercise (Zajenkowski et al., 2014; Basso and Suzuki, 2017) is attributable to opioid endogenous activity in the central and peripheral nervous system stimulated by physical activity (Anderson and Shivakumar, 2013; Fuss et al., 2015; Szabo et al., 2019). Beyond these chemical causes, physical activity is also cited by many as a valuable way of coping with stress (Steptoe et al., 1998; Cairney et al., 2014). Physical activity may reduce tension, induce relaxation, provide a distraction, and lessen rumination over stressors, which are all considered effective means for coping with stress (Hilt and Pollak, 2012; Sharon-David and Tenenbaum, 2017). A single bout of exercise can be enough to bring about a positive change, with research suggesting that as little as 10 min of aerobic exercise can have a positive effect on mood, reduced stress, and perceived energy levels (Rudolph and Butki, 1998; Loy et al., 2013, 2018).

United Kingdom participants reported significantly poorer physical health “during the last week” than did participants from Finland. While we could look to demographic factors to explain the poorer outcomes of the United Kingdom sample (e.g., they were the second oldest of the international groupings (*M*age = 38.6, *SD* = 12.78), predominantly female (80%) and married (64%), these demographic characteristics are shared by Finland, and therefore do not hold as an explanation. The factor differentiating Finland from the United Kingdom, and thus having possible influence, was the lockdown situation, with only 2% of Finnish participants perceiving a lockdown situation, as compared to 87% of those in the United Kingdom. While the United Kingdom had a mandated lockdown, it is of note that 13% of those from the United Kingdom did not self-define as such. This presents an example of where the measurement of perceived lockdown status offers a more precise indicator of personal experience than applying the Government mandated lockdown status of the respective country, providing support for the appropriateness of our approach. Further evidence for the possible influence of lockdown derived from interactions between country and physical activity, which indicated an effect on the reported wellbeing of participants, but no effect on the other outcome variables. Participants from Finland reported positive outcomes regarding perceived changes in wellbeing, along with those from the Philippines. In contrast, Italian participants reported the lowest wellbeing outcomes, and the United Kingdom the second lowest. The outcomes for Finland, the United Kingdom, and Italy partially align with lockdown status. While a low percentage of those in Italy perceived themselves to be in lockdown, it is worth noting that they were the earliest European country to enter lockdown and at the time of data collection were experiencing an easing of restrictions. Filipino participants did not align with the lockdown and wellbeing trend as a high proportion perceived themselves to be in lockdown (84%) at the time of data collection, yet still reported positive outcomes in terms of wellbeing. It is possible that the low average age (*M*age = 29.61, *SD* = 10.71) of the Filipino sample, and the high proportion (82%) of single status individuals included, denote a population with fewer responsibilities than those of the age to be in full time employment and with parenting and/or other caring responsibilities. In support of this finding, in previous quarantine situations, it has been found that undergraduate students experienced no adverse outcomes in terms of stress or general mental health (Wang et al., 2011).

Poorer perceived outcomes for mental health were observed in Latin American participants, compared with South Koreans who were significantly less affected. Again, this reflects lockdown status, with those in lockdown perceiving the most adverse outcomes. An early study in China recorded higher levels of anxiety and depression for those involved in quarantine situations, compared with those unaffected by quarantine (Lei et al., 2020). Of further note, countries with poorer outcomes were more severely affected by the virus at the time of data collection. Across the international groupings sampled, Latin America and the United States had high number of total cases and COVID-19 related deaths, while the lowest cases and deaths were observed in Finland and South Korea (Roser et al., 2020). South Korea also falls into the category of successfully managing the pandemic, if the principle of “flattening the curve” is used as an indicator (Leslie et al., 2020). Conversely, this indicator would denote Italy, United States, United Kingdom (Leslie et al., 2020), and Spain (Baniamin et al., 2020) as under-performing as they faced a long period of exponential growth. Perceiving containment of the virus may have provided reassurance to the Korean participants and may explain why they did not report such adverse outcomes. A key stressor reported in previous quarantine situations was a lack of information and lack of clarity from health and government officials (Brooks et al., 2020). In respect of sleep and eating, participants from the Philippines reported significantly higher mean scores compared to most other countries. The country experiencing the least change in eating behavior was South Korea. Again, these findings follow the pattern of those in lockdown reporting more change. No significant differences between countries were observed for changes in body weight. However, it is possible that grouping “by country” has masked a moderating effect resulting in large variations in reporting within countries.

In summary, the countries least affected by adverse outcomes could be characterized by not being in lockdown status and experiencing lower mortality and prevalence of COVID-19 at the time of data collection. It is of note that prior experience of epidemics did not consistently result in improved outcomes, as may be the case when applying the COR theory (Hobfoll, 2011). South Korea recently experienced the MERS-CoV epidemic, and the comparatively favorable outcomes observed in the present study may suggest that this provided valuable opportunity to identify and establish resources facilitative of coping with the subsequent pandemic. Conversely, experience of the Zika Virus in Latin America does not seem to have offered the same protective mechanisms to participants in the present study. It could be argued that South Korea’s response to MERS-CoV shared some similarities with responses to COVID-19, for example, quarantine of those with the virus, and restrictions on travel (World Health Organization, 2015). While the Zika virus was declared a public health risk of international concern, no restrictions on individuals or travel were deemed to be justifiable (World Health Organization, 2016). Therefore, it could be argued that the experience of the Zika epidemic was not sufficiently similar to COVID-19 for individuals to have opportunity to utilize any prior coping experience.

When comparing mental health across the four age groups, participants in the oldest category (>43 years of age) reported being least affected by personal and emotional problems than those in the youngest groups. The finding is interesting considering that older people have been disproportionally affected by COVID-19, with fatality rates five times higher than the global average for individuals aged 80 or older (United Nations, 2020). This result, however, is in line with a study of 1,679 Dutch participants aged 65 or older, who did not experience a decrease in mental health during the COVID-19 pandemic compared to 6 months prior (van Tilburg et al., 2020). Studies in China (Liu et al., 2020), Italy (Di Renzo et al., 2020), and Spain (Rodríguez-Rey et al., 2020a) also indicate a higher level of anxiety among younger participants during pandemic.

Working situation was a significant covariate in explaining the variance in outcomes. Overall, only 25.6% of participants reported a similar workload during the pandemic. Homeworking, experienced by 34.6% of respondents, may also have signified a change in the way of working for many. While a shift to homeworking may bring some relief from stress through less time spent commuting, remote workers may experience conflicting demands on time (de Menezes and Kelliher, 2011). During lockdown, this was exacerbated among those with caring and home-schooling responsibilities. A minority of 8.7% were affected by job loss; which is well documented to have a detrimental impact on mental health (Paul and Moser, 2009), and has also been demonstrated in previous viral epidemics (Taylor et al., 2008).

When considered in the context of the conservation of resources theory (Hobfoll, 2011), physical activity may be considered a beneficial coping resource utilized in preventing or ameliorating the effects of stress. Support for this notion is offered by findings of the present study which indicate that irrespective of age and country of residence, participants self-reporting reduced physical activity typically experienced poorer physical health and mental health as rated ‘over the past week’ along with reduced wellbeing. Reduced exercise also associated with perceptions of weight gain and decreased sleep. Associations between increased exercise and perceived changes in eating were variable dependent on age and country. These findings endorse the policy of advocating physical activity as a means of generating and maintaining resources combative of stress and protective of health during the pandemic (Zhou and Lin, 2016).

When examining physical activity behaviors during COVID-19, it was apparent that while many individuals perceived a large-to-moderate increase in physical activity, many also perceived a large-to-moderate decrease. This is to be expected, as existing research indicates that social isolation is associated with an increase in sedentary behaviors (Tully et al., 2019), and affiliation is a common motive for physical activity participation (Estabrooks and Carron, 2000). In addition, the closure of gyms, parks, and recreational facilities likely had a negative influence on physical activity for many (Arora and Grey, 2020). As such, for individuals to maintain or increase exercise during the pandemic, both an intent and the means to increase exercise are necessary. A recommendation is that to engender intent, and facilitate means, physical activity guidelines for indoor and outdoor exercise are developed that take into account government containment measures and meet affiliation needs in alternative ways. These recommendations should be widely disseminated via a range of media and supplemented with information on the acute and chronic benefits of physical activity. For example, older adults who may be shielding, and thus face constraints in undertaking outdoor exercise, may use technology to support indoor physical activity. Anecdotal evidence suggests that video communication technologies (e.g., zoom) have been widely used to facilitate the continuation of exercise classes (e.g., Pilates) during the pandemic, presenting a social opportunity also. In a further illustrative example of the use of technology, de Morais et al. (2020) found acute mood benefits for older adults who followed 60 min of Xbox exercise. Where it is not physically or practically possible to accommodate longer durations of physical activity, individuals may follow “exercise snacking,” a term that describes short bouts (e.g., 2–10 min) of physical activity undertaken across the day (Perkin et al., 2019). Two papers examining the psychological consequences of COVID-19, independently concluded that suggestions for the effective regulation of emotions should be provided during times of uncertainty, such as COVID-19, which presents the potential for increased stress (Brooks et al., 2020; Pietrabissa and Simpson, 2020). In this regard, based on findings from the present study, we advocate the use of physical activity as a resource for coping and emotion regulation. By recommending and disseminating credible resources, such as those provided by World Health Organization (WHO), n.d., practitioner and academic communities may facilitate a means of exercise across a broader range of socio-demographic populations.

Limitations of the present study have emerged due to the practicalities of undertaking research during a pandemic; those being recruitment trends associated with online data collection and gathering data via self-report rather than direct measurement. The use of a web-based survey to recruit participants meant reliance individuals coming across, then completing the survey on a voluntary basis. The distribution of the survey links via social networking sites such as Facebook, Twitter, and LinkedIn may have also contributed to the skewed demographic composition of participants. For example, 77% of participants were aged 18 to 44, and only 2% aged >65. It is likely that the low recruitment of older adults (65 years and above) reflects patterns of technology use typically evidenced among older and younger adults. Research indicates that younger adults (18–28 years) use the internet fairly frequently across a wide variety of domains, while usage by older adults (65–90 years) is typically limited to communication or searching for information about community, health, news, and travel (Olson et al., 2011). This may explain why few older adults took part in the present study. Caution must be exercised in generalizing findings to older adults, and the need to consider other means of recruiting participants in future research is highlighted.

Collection of self-reported online data has the potential of introducing reporting and recall bias. For example, compared to device measured data, Pursey et al. (2014) found web-based self-reported weight to be underestimated (by −0.55 kg, *SD* = 2.03), Lauderdale et al. (2008) found sleep to be over-reported (1.3 and 0.3 hours respectively for participants sleeping 5 and 7 h), and Cerin et al. (2016) found that participants over-reported (929 min⋅wk^–1^) total physical activity over a week. However, evidence shows that there is moderate to high agreement between self-reported and measured data, therefore, online self-report can be a valid method of collecting data (Pursey et al., 2014). This is particularly the case where device measurement is impractical or prohibited, as in the case of the present study. Despite the limitations of online recruitment and data collection, it is worth noting that web-based survey administration allows truly voluntary participation, with the confounding of data due to extrinsically motivated measure completion less likely to occur (Reips, 2006), and in the case of social distancing restrictions, it was the only form of administration viable.

Wellbeing outcomes could not consistently be explained by the staging of containment measures. The two countries experiencing the virus for the longest duration were South Korea, in which comparatively better outcomes were observed, and Italy, where outcomes were poorer. This suggests that variables other than staging of response may be important in determining the outcomes under investigation. Future research may further investigate the impact of the phase of response on wellbeing indices by utilizing a phased approach which takes into account the containment policy of participating countries. This may be done in tandem with a long-term follow up of impact on wellbeing, so that we can better understand the trajectory of wellbeing outcomes in response to pandemic development.

Further research could examine how the effects of physical activity on wellbeing indicators vary according to other demographic variables, which the data did not allow for in the present study. For example, COVID-19 has had a disproportionately high impact on Black, Asian and minority ethnic groups, and low-income populations (Khalatbari-Soltani et al., 2020), which may have bearing on wellbeing. In addition, there is evidence of gender inequalities whereby women are assuming more responsibility for unpaid caring, childcare, and home-schooling (Landivar et al., 2020), suggesting poorer outcomes may be expected for women when compared to men.

It was found that younger participants (18–23 years) who reported increased physical activity also ate more, compared to those with reduced physical activity during the pandemic. It is worth noting that these participants reported the highest unemployment ratings (up to 35%). Interestingly, younger Italian participants (18–30 years) reported eating more to manage their anxiety during lockdown (Di Renzo et al., 2020). While it is possible that younger participants were engaging in compensatory eating (West et al., 2017), it is also possible that this group may be less likely to have caring responsibilities and occupational stressors. When coupled with a higher unemployment rate, this may result in an increased availability of time to engage with activities such as eating and exercise as self-soothing activities targeted at helping them to cope with the pandemic situation. Given that the perception of lockdown status and working situation were both significant covariates, the potential for compensatory eating and potentially emotional eating could be higher among younger participants (West et al., 2017; Di Renzo et al., 2020) and could be associated with the higher level of personal and emotional difficulties reported. The impacts of COVID-19 and its associated lockdown restrictions could manifest themselves differently leading to either an accumulation or loss of resources (Hobfoll, 2011), depending on environmental factors and personal circumstances, such as age and psychological coping mechanisms.

At the time of writing, the potential of a second lockdown and the reality of renewed localized containment measures could bring about further changes in health behavior and wellbeing as the pandemic unfolds. Future research might aim to better understand the causal pathways behind health behaviors and their long-term impact on wellbeing. It is plausible that participants initiated a range of coping strategies that impacted positively upon their wellbeing. Knowing what participants are doing to ameliorate the impact of the pandemic on their wellbeing, and what effect this has on wellbeing indicators, can offer insight into the development of interventions and activities recommended for use. Therefore, there is scope to explore the means by which individuals seek to cope with the pandemic using qualitative methodologies.

## Data Availability Statement

The raw data supporting the conclusions of this article will be made available by the authors, without undue reservation.

## Ethics Statement

This study was carried out in accordance with ‘the ethical principles of research in the humanities and social and behavioral sciences and proposals for ethical review’ drafted by the National Advisory Board on Research Ethics in Finland (TENK). This study received ethical approval from the University of Wolverhampton ethics committee (Unique code: 01/20/AF1/UOW). In accordance with the Declaration of Helsinki, all participants gave informed consent, after anonymity and confidentiality were assured.

## Author Contributions

MR, TD, and C-HC-W: conceptualization and methodology. C-HC-W: data management. MR and C-HC-W: data curation. MR and CR: formal analysis. MR, TD, C-HC-W, and WN: writing – original draft. MR, TD, C-HC-W, WN, JC, JF-M, YC, and CR: writing – review and editing. All authors contributed to the article and approved the submitted version.

## Conflict of Interest

The authors declare that the research was conducted in the absence of any commercial or financial relationships that could be construed as a potential conflict of interest.

## Footnotes

1 We aimed at recruiting participants from single Spanish-speaking countries in South America (e.g., Chile) and Central America (e.g., Costa Rica) as well as Mexico, through associations and institutional contacts (e.g., universities, colleges of psychologists). Due to low participation from single countries (e.g., Chile [*n* = 48], Mexico [*n* = 29], and Costa Rica [*n* = 25], were most represented), all participants were included within the same group, which we refer to as Latin America.

2 Demographic characteristics of participants (*n* = 1,140) can be found in the Supplementary Table S1.
