# Supplementary material for: A Cross-Cultural Exploratory Study of Health Behaviors and Wellbeing During COVID-19
Source: Front Psychol. 2021 Jan 12;11:608216. doi: 10.3389/fpsyg.2020.608216 (PMC7835515; doi:10.3389/fpsyg.2020.608216)
Supplement: Supplementary file 1 [file Data_Sheet_1.PDF]

Supplementary Table S1. Participants' demographic information, restrictions, and socioeconomic characteristics.

		UK (N=230)	South Korea (N=204)	Finland (N=171)	Philippines (N=132)	Latin America (N=124)	Spain (N=112)	Italy (N=80)	North America (N=87)
Age	Min	18	19	18	18	18	21	19	19
	Max	76	60	75	66	70	72	66	73
	M	38.61	27.01	34.57	29.61	30.60	44.63	33.98	33.77
	SD	12.78	8.334	13.40	10.715	12.13	10.49	12.732	14.171
Gender	Female	185 (80)	129 (63)	153 (90)	69 (52)	67 (54)	82 (73)	57 (71)	69 (79)
	Male	36 (16)	63 (31)	17 (10)	59 (45)	57 (46)	30 (27)	19 (24)	18 (21)
	Other	0 (0)	2 (1)	0 (0)	1 (1)	0 (0)	0 (0)	0 (0)	0 (0)
	Not reported	9 (4)	10 (5)	1 (1)	3 (2)	0 (0)	0 (0)	4 (5)	0 (0)
Working situation	Similar load	52 (23)	115 (56)	50 (29)	17 (13)	13 (10)	31 (28)	19 (24)	19 (22)
	Increased load	40 (17)	21 (10)	31 (18)	17 (13)	27 (22)	26 (23)	10 (13)	11 (13)
	Decreased load	22 (10)	54 (26)	23 (13)	15 (11)	33 (27)	19 (17)	15 (19)	8 (9)
	Homeworking	83 (36)	31 (15)	102 (60)	52 (39)	55 (44)	27 (24)	25 (31)	24 (28)
	Laid off	29 (13)	5 (2)	10 (6)	12 (9)	2 (2)	18 (16)	3 (4)	16 (18)
	Unemployed	30 (13)	9 (4)	14 (8)	43 (33)	27 (22)	12 (11)	16 (20)	16 (18)
Relationship status	Single	67 (29)	168 (82)	57 (33)	111 (84)	82 (66)	30 (27)	43 (54)	40 (46)
	Married/partnership	148 (64)	31 (15)	105 (61)	16 (12)	34 (27)	69 (62)	32 (40)	39 (45)
	Divorced	10 (4)	1 (0)	5 (3)	2 (2)	8 (6)	10 (9)	1 (1)	5 (6)
	Widowed	2 (1)	1 (0)	1 (1)	1 (1)	0 (0)	2 (2)	1 (1)	1 (1)
	Prefer not to say	3 (1)	2 (1)	3 (2)	2 (2)	0 (0)	1 (1)	3 (4)	2 (2)
	Not reported	0 (0)	1 (1)	0 (0)	0 (0)	0 (0)	0 (0)	0 (0)	0 (0)
Lockdown	Yes	200 (87)	5 (3)	4 (2)	111 (84)	112 (90)	104 (93)	18 (23)	56 (64)
	No	28 (12)	190 (93)	166 (97)	19 (14)	12 (10)	8 (7)	61 (76)	29 (33)
	Do not know	2 (1)	9 (4)	1 (1)	2 (2)	0 (0)	0 (0)	1 (1)	2 (2)
Household number members	Living alone	29 (13)	48 (24)	50 (29)	16 (12)	11 (9)	19 (17)	13 (16)	9 (10)
	With one other	63 (27)	31 (15)	66 (39)	13 (10)	24 (19)	28 (25)	26 (33)	23 (26)
	With two or more people	138 (60)	125 (61)	55 (33)	103 (78)	89 (72)	65 (58)	41 (51)	55 (63)
Household type	Apt. no balcony/terrace/garden	18 (8)	89 (44)	29 (17)	18 (14)	11 (9)	32 (29)	5 (6)	5 (6)
	Apt. with balcony/garden	16 (7)	37 (18)	75 (43)	16 (12)	23 (19)	59 (53)	44 (55)	18 (21)
	Det./family house no garden/yard	3 (1)	23 (11)	66 (39)	32 (24)	19 (15)	4 (4)	4 (5)	2 (2)
	Det./family house with garden/yard	173 (75)	19 (9)	1 (1)	58 (44)	71 (57)	17 (15)	25 (31)	61 (70)
	Other	20 (9)	36 (18)	0 (0)	8 (6)	0 (0)	0 (0)	2 (3)	1 (1)

Note. Apt. = Apartment; Det. = detached house; values in parenthesis represent percentages.

Supplementary Table S2. Participants' reported scores in studied variables for different countries and age groupings.

	United Kingdom (N=230)															
	18-23 years old (N= 35)				24-30 years old (N= 36)				31-43 years old (N= 75)				>43 years old (N= 84)			
Variable	*M*	*SD*	*SK*	*K*	*M*	*SD*	*SK*	*K*	*M*	*SD*	*SK*	*K*	*M*	*SD*	*SK*	*K*
Physical health during last week	3.23	1.11	0.47	-0.55	3.56	1.25	0.01	-0.68	3.60	1.04	0.58	-0.21	3.68	1.08	0.09	-0.27
Emotional impact during last week	3.40	1.09	-0.31	-0.83	3.03	1.16	-0.17	-0.79	2.36	1.02	0.08	-1.12	2.12	1.00	0.42	-0.92
Physical activity	-1.17	2.94	0.51	-0.83	0.56	3.50	-0.44	-1.19	-0.63	2.85	0.46	-0.95	0.63	3.10	-0.19	-1.07
Eating	1.57	2.34	-0.29	-1.25	1.69	2.30	-0.51	-0.60	1.40	2.25	-0.78	0.55	1.07	1.85	-0.48	1.45
Sleep	1.86	2.83	-0.99	0.34	0.19	3.06	-0.17	-0.78	0.23	2.47	-0.26	-0.68	-0.17	2.29	0.32	-0.08
Weight	1.03	2.35	-0.36	0.38	1.19	2.69	-0.03	-1.06	1.40	2.49	-0.20	-0.83	1.05	1.84	0.18	-0.30
Wellbeing	-0.60	2.56	0.50	0.40	-1.17	2.65	0.77	0.28	-0.93	2.27	0.64	-0.02	-0.27	2.07	0.42	-0.10
	**South Korea (N=204)**															
	**18-23 years old (N= 97)**				**24-30 years old (N=61)**				**31-43 years old (N= 32)**				**>43 years old (N= 14)**			
	M	*SD*	*SK*	*K*	*M*	*SD*	*SK*	*K*	*M*	*SD*	*SK*	*K*	*M*	*SD*	*SK*	*K*
Physical health during last week	3.75	1.23	0.11	-0.34	3.75	1.04	0.24	0.22	3.63	0.83	0.47	-0.79	3.21	0.70	1.25	2.88
Emotional impact during last week	2.13	1.18	0.55	-1.21	2.13	1.22	0.60	-1.05	1.50	0.72	1.11	-0.08	1.43	0.85	2.44	6.48
Physical activity	0.24	2.96	-0.07	-0.90	0.26	2.29	0.11	-0.01	-0.41	2.56	-0.04	-0.72	0.29	1.98	-0.32	0.47
Eating	0.33	2.12	0.04	0.19	0.61	1.72	1.11	1.83	0.47	1.27	-0.58	4.74	0.86	1.41	1.07	0.47
Sleep	0.46	2.75	0.08	-0.92	0.28	2.22	0.32	-0.26	0.34	1.45	0.77	0.62	0.00	1.36	0.64	0.90
Weight	0.69	2.15	0.08	-0.06	0.90	1.98	0.07	0.64	0.84	1.94	0.46	-0.19	1.64	1.82	0.18	-0.73
Wellbeing	-0.34	2.36	0.41	0.38	-0.26	1.78	-0.03	1.06	0.13	2.15	-0.07	-0.05	0.43	1.95	-0.49	-0.53
	**Finland (N=171)**															
	**18-23 years old (N= 39)**				**24-30 years old (N=50)**				**31-43 years old (N= 41)**				**>43 years old (N= 41)**			
	M	*SD*	*SK*	*K*	*M*	*SD*	*SK*	*K*	*M*	*SD*	*SK*	*K*	*M*	*SD*	*SK*	*K*
Physical health during last week	4.26	1.07	-0.41	-0.16	4.40	0.95	-0.45	0.28	4.02	0.96	-0.41	1.32	4.00	0.87	0.00	-0.45
Emotional impact during last week	2.49	1.02	0.27	-0.38	2.40	0.90	0.31	-0.60	2.27	0.87	0.40	-0.30	2.24	0.89	-0.06	-0.99
Physical activity	0.62	2.55	-0.40	-0.22	0.76	2.09	-0.02	-0.41	-0.20	2.39	-0.12	-0.89	0.51	2.27	0.44	-0.80
Eating	-0.03	2.32	0.63	0.03	1.02	1.57	-0.20	1.23	1.07	1.74	0.51	-0.02	1.24	1.32	0.35	-1.23
Sleep	0.59	1.86	0.02	0.36	1.10	1.82	-0.13	-0.70	0.32	1.89	0.22	0.56	0.20	1.91	-0.09	0.30
Weight	0.41	2.19	0.26	0.38	0.44	1.64	0.37	1.46	0.80	2.05	0.64	-0.28	1.12	1.82	0.41	-0.40
Wellbeing	-0.28	2.18	0.16	-0.03	0.28	2.22	-0.29	-0.77	-0.22	2.19	0.19	0.49	-0.20	1.75	1.34	2.22

	**Philippines (N=132)**															
	**18-23 years old (N= 52)**				**24-30 years old (N=31)**				**31-43 years old (N= 35)**				**>43 years old (N= 14)**			
	M	*SD*	*SK*	*K*	*M*	*SD*	*SK*	*K*	*M*	*SD*	*SK*	*K*	*M*	*SD*	*SK*	*K*
Physical health during last week	3.75	1.19	0.29	-0.65	3.39	1.17	-0.04	0.00	3.57	1.01	-0.12	-1.00	3.86	0.95	0.95	0.34
Emotional impact during last week	3.25	0.88	-0.34	-0.44	3.23	0.99	0.17	-1.05	2.91	0.95	-0.69	-0.23	2.64	0.84	-0.07	-0.18
Physical activity	0.63	3.09	-0.20	-1.24	-0.32	3.28	-0.05	-1.37	0.34	3.08	-0.29	-1.11	0.79	3.07	-0.58	-0.99
Eating	2.10	2.43	-1.13	1.29	2.03	1.96	-0.68	0.37	1.14	2.44	-0.73	0.12	1.21	1.85	0.23	-0.49
Sleep	2.62	2.47	-1.57	2.54	1.61	2.49	-0.71	0.13	0.34	2.97	-0.41	-0.93	1.64	2.13	0.43	-1.23
Weight	1.13	2.67	-0.38	-0.73	1.29	2.75	-0.10	-1.22	0.80	2.78	-0.07	-1.11	0.50	1.91	0.08	-1.06
Wellbeing	0.56	2.70	0.05	-0.85	0.26	2.66	-0.24	-0.56	0.51	2.45	-0.08	-0.58	0.21	1.89	0.92	-0.15
	**Latin America (N=124)**															
	**18-23 years old (N= 50)**				**24-30 years old (N=30)**				**31-43 years old (N= 25)**				**>43 years old (N= 19)**			
	M	*SD*	*SK*	*K*	*M*	*SD*	*SK*	*K*	*M*	*SD*	*SK*	*K*	*M*	*SD*	*SK*	*K*
Physical health during last week	3.78	1.09	0.26	0.07	3.90	1.03	0.21	-0.32	4.32	1.18	-0.85	1.07	4.74	0.65	0.31	-0.51
Emotional impact during last week	3.54	0.86	-0.33	-0.51	3.13	1.11	-0.77	-0.29	2.96	1.10	0.09	-0.58	2.58	1.02	0.65	0.49
Physical activity	-0.28	3.10	0.04	-1.17	-0.23	2.50	0.13	-0.74	0.24	2.85	-0.57	-0.41	-0.26	2.90	0.11	-0.96
Eating	1.62	1.72	0.00	-0.84	1.27	2.12	0.35	-0.92	0.68	2.25	-0.16	-0.44	1.00	1.37	-0.29	-0.22
Sleep	0.42	3.10	0.16	-1.23	0.50	3.01	0.00	-0.84	-0.28	2.30	1.18	0.83	-0.26	2.21	0.10	0.27
Weight	0.74	1.83	0.59	0.88	1.17	2.18	-0.04	0.37	0.88	2.44	-0.07	0.26	0.58	1.57	-0.54	0.24
Wellbeing	-0.96	2.17	0.35	0.14	-0.63	2.28	0.80	0.24	-0.52	2.18	0.50	0.25	-0.05	1.35	0.26	-0.83
	**Spain (N=112)**															
	**18-23 years old (N= 4)**				**24-30 years old (N=9)**				**31-43 years old (N= 30)**				**>43 years old (N= 69)**			
	M	*SD*	*SK*	*K*	*M*	*SD*	*SK*	*K*	*M*	*SD*	*SK*	*K*	*M*	*SD*	*SK*	*K*
Physical health during last week	4.00	0.82	0.00	1.50	4.22	0.97	0.50	-0.01	3.73	0.94	-0.47	2.07	4.07	0.94	-0.15	1.01
Emotional impact during last week	2.25	1.50	0.37	-3.90	3.11	1.17	-0.27	0.54	3.13	0.82	-0.26	0.80	2.52	1.24	0.23	-1.17
Physical activity	-1.50	1.29	0.00	-1.20	-0.22	4.09	0.21	-1.64	-0.53	2.34	0.10	-0.91	0.57	2.90	0.01	-1.00
Eating	0.00	2.31	0.00	-6.00	2.00	2.35	0.15	-1.84	0.93	1.70	-0.07	-0.26	0.87	1.79	0.52	0.71
Sleep	0.75	3.30	-1.56	2.17	0.22	3.42	0.14	-0.79	0.63	2.14	0.11	0.24	0.22	2.22	0.37	-0.60
Weight	-0.50	1.73	0.00	-6.00	1.56	3.17	-0.36	-0.75	0.87	1.74	0.56	-0.20	0.83	1.76	0.11	0.56
Wellbeing	1.25	1.71	-0.75	0.34	-0.78	2.54	1.69	3.04	-1.07	2.00	0.99	2.28	0.00	2.09	0.68	-0.30

	North America (N=87)															
	18-23 years old (N= 26)				24-30 years old (N=21)				31-43 years old (N= 21)				>43 years old (N= 19)			
	M	*SD*	*SK*	*K*	*M*	*SD*	*SK*	*K*	*M*	*SD*	*SK*	*K*	*M*	*SD*	*SK*	*K*
Physical health during last week	3.73	1.15	0.58	-0.08	3.86	1.20	0.11	-0.62	4.05	1.12	0.14	-0.90	4.42	0.96	0.25	-0.69
Emotional impact during last week	2.81	1.02	-0.07	-0.22	2.95	0.92	0.10	0.35	2.48	1.17	0.17	-0.57	2.58	1.02	0.29	0.62
Physical activity	-0.12	3.60	0.04	-1.47	0.24	3.36	-0.01	-1.00	-0.76	2.86	0.29	-0.77	0.42	2.17	-0.14	-0.12
Eating	1.15	2.95	-0.46	-0.52	1.33	1.96	-1.09	0.45	0.71	1.82	0.47	0.76	1.00	1.94	0.41	-1.25
Sleep	0.65	3.70	-0.37	-1.35	1.10	2.55	-0.18	-0.87	0.62	2.62	0.05	-0.81	1.11	2.05	0.45	-1.07
Weight	0.46	1.50	-0.65	2.81	0.57	2.36	0.07	-0.16	1.62	2.22	0.02	-0.70	0.63	2.14	0.23	-0.37
Wellbeing	-0.35	2.43	0.33	-0.36	-0.76	2.34	0.30	0.22	-0.33	2.33	0.11	0.41	-0.21	1.62	-0.15	0.80
	Italy (N=80)															
	18-23 years old (N= 14)				24-30 years old (N=33)				31-43 years old (N= 13)				>43 years old (N= 20)			
	M	*SD*	*SK*	*K*	*M*	*SD*	*SK*	*K*	*M*	*SD*	*SK*	*K*	*M*	*SD*	*SK*	*K*
Physical health during last week	3.71	1.14	0.29	-0.12	3.48	1.23	-0.29	-0.03	3.62	0.87	0.03	-0.32	3.90	0.91	0.68	-0.35
Emotional impact during last week	2.36	1.15	0.94	0.83	2.45	1.06	0.79	0.38	2.00	1.29	1.37	1.23	1.70	0.73	0.55	-0.83
Physical activity	0.71	2.61	0.61	-1.10	0.27	3.69	-0.13	-1.48	0.00	2.97	0.07	-0.58	-0.65	2.72	-0.24	-1.28
Eating	1.36	1.86	0.05	-1.59	1.09	2.64	-0.54	0.01	1.46	1.56	-0.77	0.28	1.05	1.64	0.23	-0.90
Sleep	0.50	2.35	-0.50	1.90	-0.03	2.57	-0.40	-0.34	-0.38	2.63	0.33	-0.55	-0.95	1.70	-0.44	-1.04
Weight	0.93	2.20	-0.10	-0.41	0.82	2.35	-0.38	0.11	1.69	2.36	-1.05	1.58	1.05	2.26	0.14	-0.59
Wellbeing	-1.00	2.39	0.08	-0.84	-1.48	2.45	0.63	0.57	-0.23	2.24	0.65	-0.83	-0.75	1.62	0.13	0.62

Supplementary Table S3. Bivariate correlations for the studied variables for the whole sample (*N* = 1,131)

	1	2	3	4	5	6
1. Physical health during last week						
2. Emotional impact during last week	-0.34*					
3. Physical activity	0.31*	-0.24*				
4. Eating	-0.07	0.15	-0.04			
5. Sleep	0.09	0.01	0.14	0.15		
6. Weight	-0.21*	0.13	-0.25*	0.54**	0.03	
7. Wellbeing	0.35*	-0.31*	0.42**	-0.09	0.18	-0.20*

Note. *Low correlations, **moderate correlations. The magnitude of correlation coefficients were interpreted according to Zhu's (2012) suggestion, 0–0.19 = no correlation, 0.20–0.39 = low correlation, 0.40–0.59 = moderate correlation, 0.60–0.79 = moderately high correlation, and > 0.80 = high correlation [Zhu, W. (2012). Sadly, the earth is still round ($p < 0.05$). *Journal of Sport and Health Science, 1*(1), 9–11. https://doi.org/10.1016/j.jshs.2012.02.002]

Supplementary Figure S1. Means and standard deviations of the reported physical health (A) and impact on emotional health (B) during the previous week, and reported changes in physical activity (C), sleep (D), eating (E), weight (F), and wellbeing (G) during the COVID-19 pandemic across countries (N=1131).

A

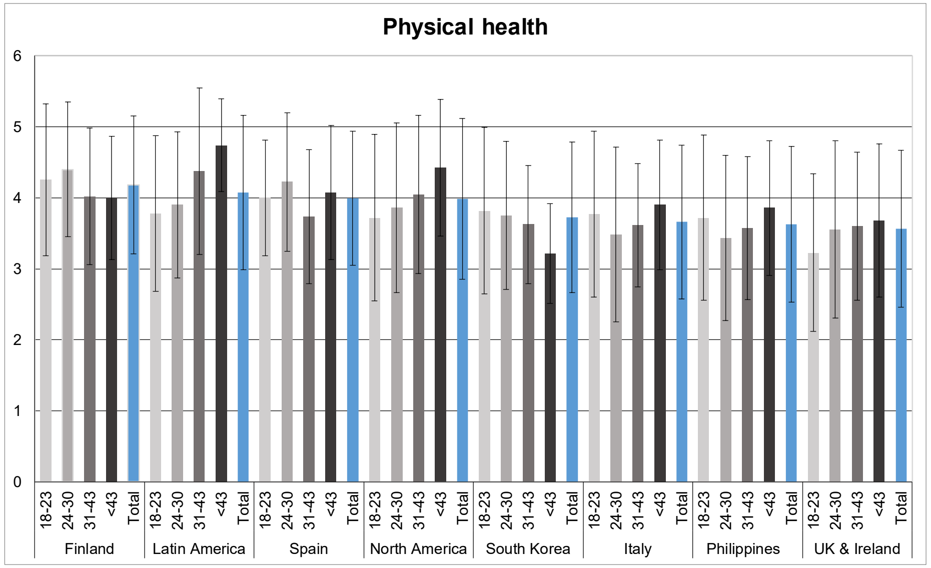

B

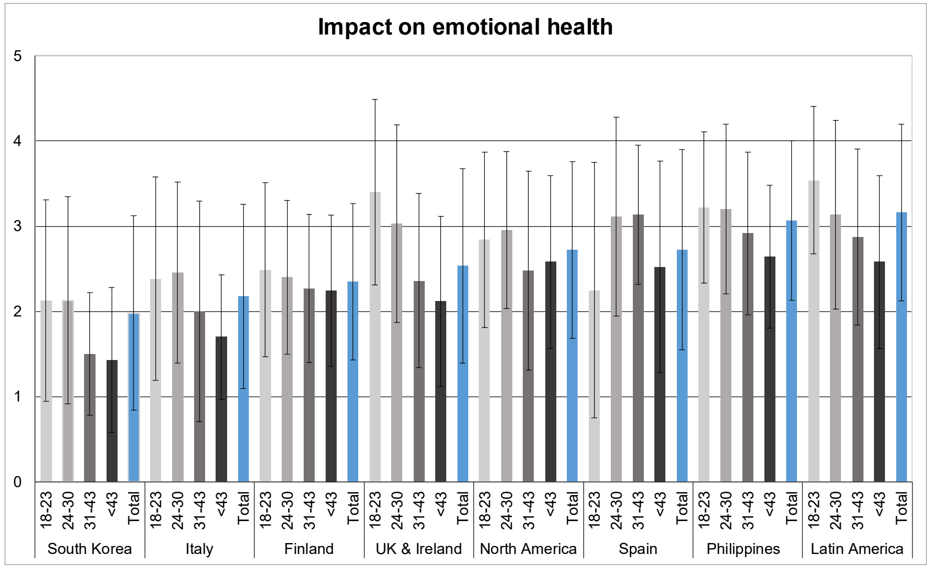

C

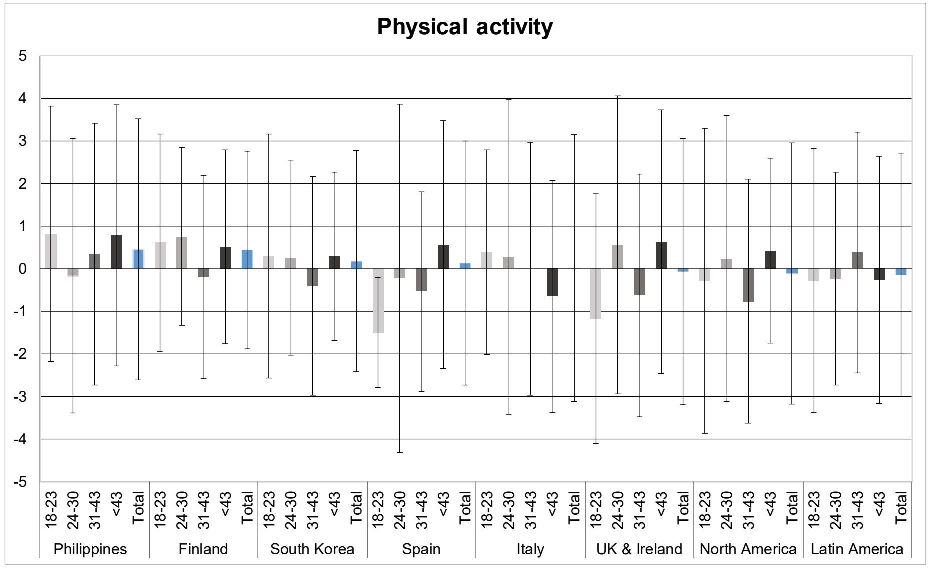

D

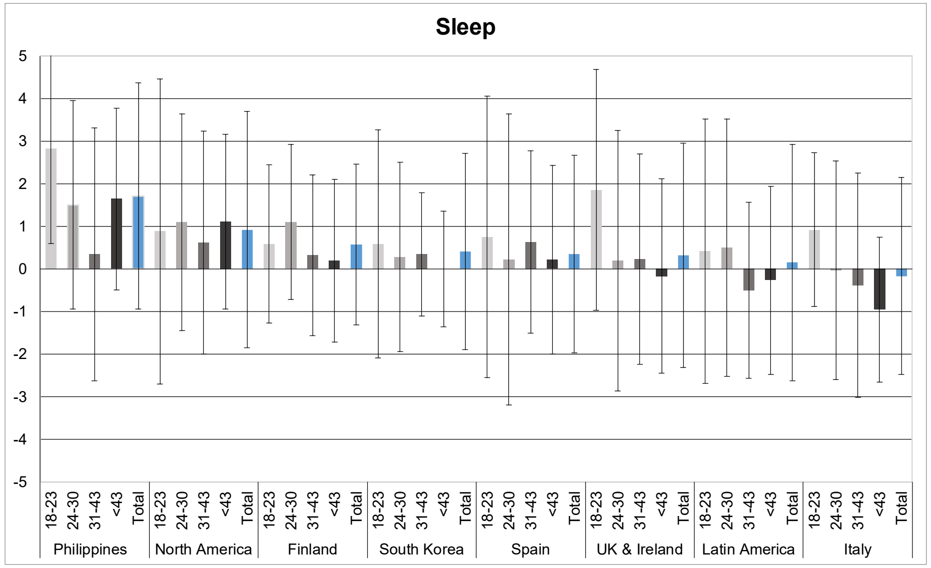

E

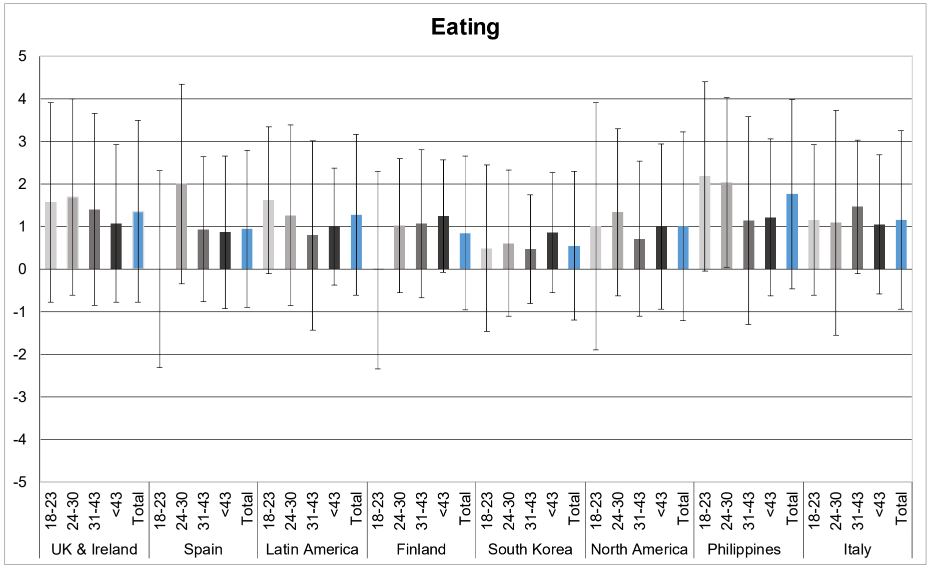
Eating
5
4
3
2
1
0
-1
-2
-3
-4
-5
18-23
24-30
31-43
<43
Total
UK & Ireland
Spain
Latin America
Finland
South Korea
North America
Philippines
Italy

F

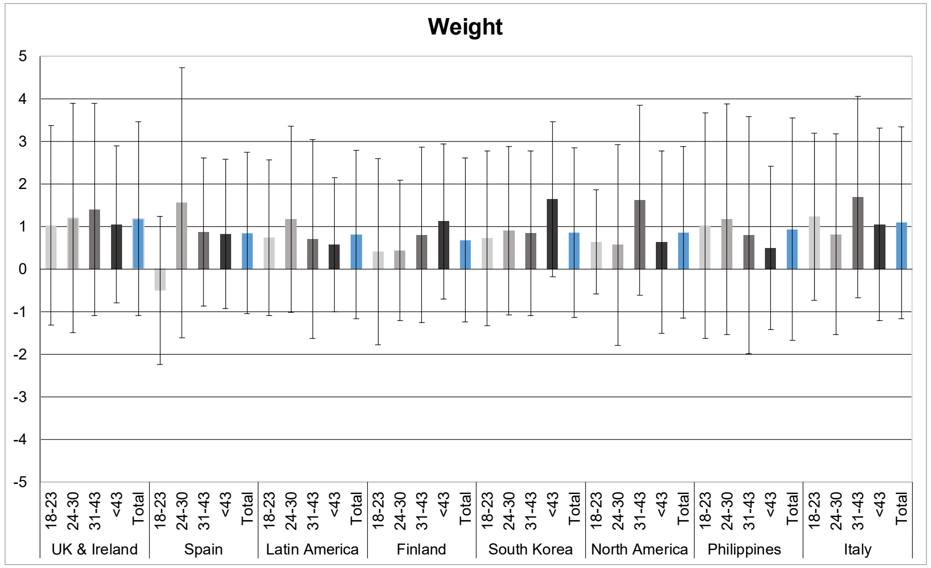
Weight
5
4
3
2
1
0
-1
-2
-3
-4
-5
18-23
24-30
31-43
<43
Total
UK & Ireland
Spain
Latin America
Finland
South Korea
North America
Philippines
Italy

G

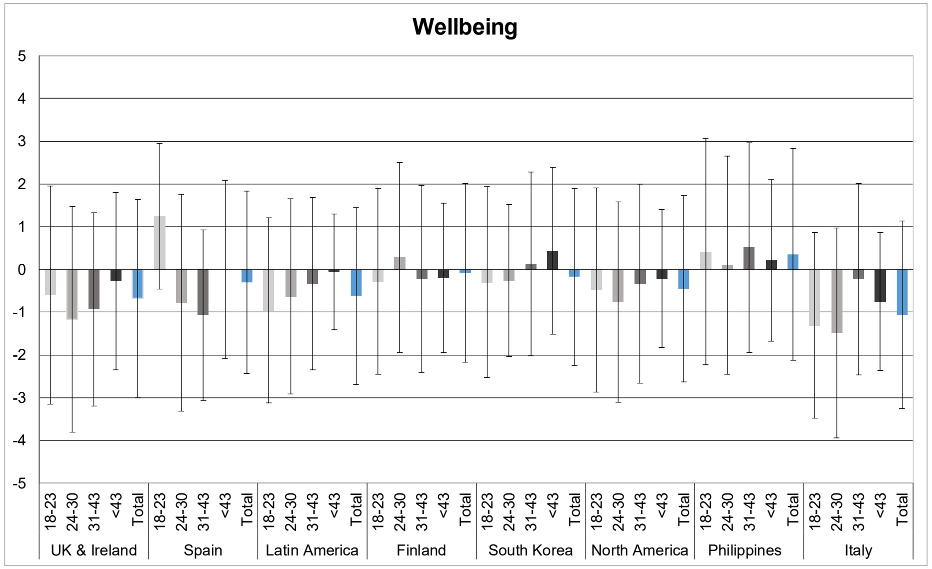
Wellbeing
5
4
3
2
1
0
-1
-2
-3
-4
-5
18-23
24-30
31-43
<43
Total
UK & Ireland
Spain
Latin America
Finland
South Korea
North America
Philippines
Italy
